# Psychosocial Experiences of Older Women in the Management of Urinary Incontinence: A Qualitative Study

**DOI:** 10.3389/fpsyg.2022.785446

**Published:** 2022-03-08

**Authors:** Sorur Javanmardifard, Mahin Gheibizadeh, Fatemeh Shirazi, Kourosh Zarea, Fariba Ghodsbin

**Affiliations:** ^1^Student Research Committee, School of Nursing and Midwifery, Ahvaz Jundishapur University of Medical Sciences, Ahvaz, Iran; ^2^Nursing Care Research Center in Chronic Diseases, School of Nursing and Midwifery, Ahvaz Jundishapur University of Medical Sciences, Ahvaz, Iran; ^3^Community Based Psychiatric Care Research Center, School of Nursing and Midwifery, Shiraz University of Medical Sciences, Shiraz, Iran; ^4^Department of Nursing, School of Nursing and Midwifery, Shiraz University of Medical Sciences, Shiraz, Iran

**Keywords:** urinary incontinence, women, psychosocial, qualitative research, older

## Abstract

**Introduction:**

Urinary incontinence is a prevalent disorder amongst older women. Identifying the psychosocial experiences of older women in disease management can improve the patient care process. Hence, the present study aimed to determine the psychosocial experiences of older women in the management of urinary incontinence.

**Methods:**

This qualitative study was conducted using conventional content analysis. The study data were collected via unstructured in-depth face-to-face interviews with 22 older women suffering from urinary incontinence selected via purposive sampling. Sampling and data analysis were done simultaneously and were continued until data saturation. The interviews were recorded, transcribed, and analyzed using the method proposed by Graneheim and Lundman.

**Results:**

The results indicated that the older people with urinary incontinence had various psychosocial experiences while living with and managing this disease. Accordingly, four main themes were extracted from the data as follows: “problem incompatibility with the disease,” “mental impasse,” “facing social restrictions,” and “concealment and social escapism.”

**Conclusion:**

The findings demonstrated that older people with urinary incontinence experienced significant psychosocial pressures while living with this disorder, which affected their psychosocial well-being. Thus, paying attention to these psychosocial experiences while supporting and taking care of these patients can positively impact their psychosocial health and quality of life.

## Introduction

Urinary incontinence has been defined as any involuntary urine leakage (Batmani et al., 2021) and is common in older adults. The prevalence of this disorder has been found to increase with age (Davis et al., 2020; Shaw and Wagg, 2021). Additionally, most epidemiological studies have reported the prevalence of urinary incontinence at 25–45% in females and 5–35% in males (Grant and Currie, 2020; Shaw and Wagg, 2021). In a study conducted in Middle Eastern countries, the prevalence of urinary incontinence was reported as 52%. In the studies conducted in different regions of Asia, the prevalence of urinary incontinence was estimated as 13% in older adults (Kaşıkçı et al., 2015; Khan et al., 2017; Batmani et al., 2021). Another study conducted in Iran indicated that the prevalence of urinary incontinence was 62.2% among women over 60 years of age (Morowatisharifabad et al., 2015).

Along with the relatively high prevalence of this problem among older adults, urinary incontinence causes changes in all aspects of older people’s lives, and it has many physical, psychological, and social effects, affecting their quality of life (Goforth and Langaker, 2016; Saboia et al., 2017; Javadifar and Komeilifar, 2018). Evidence has indicated that older adults with urinary incontinence have lower perceived health than healthy ones (Murukesu et al., 2019). In fact, due to the limitations associated with the disease, as well as the need for continuous care under different conditions, these patients encounter a variety of psychological and social problems (Higa et al., 2008; Grant and Currie, 2020), causing tension and affecting their identity, emotional balance, self-satisfaction, feeling of efficiency, social interaction, and interpersonal relationships (Afrasiabifar et al., 2010; Stickley et al., 2017).

Previous studies have shown that urinary incontinence was associated with depression, stress, and self-esteem. Accordingly, women with urinary incontinence reported significantly higher levels of depression and stress and lower self-esteem levels than those without this problem (Lee et al., 2021). On the other hand, the findings of another study on the psychosocial impacts of urinary incontinence on women’s quality of life showed that the psychosocial effects of urine leakage like anxiety, isolation, low self-esteem, and depression could worsen the symptoms of urinary incontinence. Therefore, it could adversely affect the quality of life of the affected women (Omu et al., 2020).

How the older adults cope with this disease, and attitudes about this disease are very important in the older adults and the society in which the older adults live (Avery et al., 2013) and can affect their psychosocial health as well as their quality of life (Alshammari et al., 2020). Evidence has demonstrated that older women handle these hurdles depending on their social and cultural backgrounds. In other words, each disease is caused, experienced, and managed differently by different people based on the social and cultural backgrounds of the society they live in van den Muijsenbergh and Lagro-Janssen (2006), Andersson et al. (2008), Hayder and Schnepp (2010), Gjerde (2012), Shirazi et al. (2014), Özkan et al. (2015), Heidari et al. (2021), Shakery et al. (2021). Thus, to determine the psychosocial effects of urinary incontinence on older women, their sociocultural backgrounds should be considered (Sinclair and Ramsay, 2011; Laganà et al., 2014; Alshammari et al., 2020). On the other hand, regarding each society’s sociocultural backgrounds, identifying the experiences of older women suffering from urinary incontinence, finding the strategies they use for the management of the disease, and determining the effects of those strategies on various health dimensions, particularly psychosocial health, can help nurses and other healthcare team members identify and evaluate this problem amongst older women and take measures to provide these patients with training and healthcare services. In this way, they will promote their psychosocial health and improve their quality of life (Pintos-Díaz et al., 2019). In this context, qualitative studies should be conducted based on the sociocultural features of the society where the intended older people live (Shirazi et al., 2016).

Considering Iran’s specific cultural, social, and religious backgrounds, high prevalence of urinary incontinence among older women, and lack of qualitative studies in this field, the present study aims to determine the psychosocial experiences of older women regarding the management of urinary incontinence.

## Materials and Methods

### Aim

The present study aimed to determine the psychosocial experiences of older women in the management of urinary incontinence.

### Study Design

This qualitative research was conducted via conventional content analysis. Content analysis refers to the process of perception, interpretation, and conceptualization of the inner meanings of qualitative data (Graneheim and Lundman, 2004). In this method, categories are inductively extracted from textual and verbal data (Cho and Lee, 2014).

### Participants

This study was conducted on Persian-speaking older women aged >60 years who were clinically diagnosed with one type of urinary incontinence, were suffering from the disease for at least 6 months, had no history of mental disorders, were utterly conscious, and were willing to take part in the research and share their experiences of disease management. The participants were selected via purposeful sampling from the older women referred to comprehensive health centers in Ahvaz from November 2019 until May 2020. In order to achieve maximum variation among the participants, older women with various education levels, marital statuses, and financial statuses were enrolled in the research. In other words, the researcher interviewed older adults with a suitable economic status (P: 2, 13) as well as those with moderate (P: 1, 7) or poor (P: 14, 19) economic statuses. She also interviewed older adults with a desirable literacy level (P: 9, 12) as well as illiterate ones (P: 6, 11). Interviews were also conducted with older adults living alone (P: 5, 10) as well as those living with their families (P: 3, 4) or spouses (P: 8, 17). Older women with urinary incontinence were interviewed until data saturation was achieved. After all, 22 participants were selected and interviewed.

### Data Collection

The study data were collected via unstructured in-depth face-to-face interviews in comprehensive health centers affiliated to Ahvaz Jundishapur University of Medical Sciences from November 2019 until May 2020. It should be noted that the time and place of the interviews were arranged with the participants. The interviews were begun with general questions like “what psychosocial problems has urinary incontinence created for you” and “what do you do to overcome these problems” and were continued using probing questions like “can you explain more,” “please give an example,” “how did you feel under those conditions,” and “what did you do when you encountered this problem.” The first interview was treated as preliminary and was used to identify the potential areas of interest or concern. The interviews lasted for 40–60 min and were recorded using a digital recorder (made by Sony). Purposive sampling was continued until data saturation was achieved and the collected information confirmed the previously gathered points. Overall, 24 interviews were conducted with 22 participants (two patients were interviewed twice). While analyzing the interviews, the researchers encountered some issues, which required further probing and follow-up. Therefore, two participants were interviewed twice in order to achieve richer data. After each interview, its content was transcribed verbatim in the shortest time possible.

### Data Analysis

The study data were analyzed based on the method proposed by Graneheim and Lundman (2004), which included the following stages: (1) immediate transcription of interviews, (2) reading the whole text for gaining an overall perception, (3) determination of the units of meaning and initial codes, (4) classification of the similar initial codes in more broad categories, and (5) determining the hidden content in the data (Graneheim and Lundman, 2004). Therefore, in the present study, the contents of the interviews were immediately transcribed, typed, and read several times. In this way, the meaning units were extracted from the words, sentences, and paragraphs and were coded. Afterward, a constant comparison was made, and the codes were categorized based on their similarities. Then, the initial codes were merged in order to create more abstract categories and the themes were identified. Categorization and analysis of the data were carried out using the MAXQDA 10 software.

### Ethical Considerations

After gaining permission from the Ethics Committee of Ahvaz Jundishapur University of Medical Sciences (IR.AJUMS.REC.1398.604, proposal No. NCRCCD-9825) and acquiring the necessary licenses, data collection was started. It should be noted that the participants’ oral and written informed consent was also obtained before data collection. In addition, the interviews were carried out such a way that the participants’ comfort and privacy were respected. Besides, the participants were given nicknames to ascertain the anonymity and confidentiality of their information. The participants were also assured that they could withdraw from the study without any negative impacts on their treatment processes. After all, the interviews were transcribed word by word, and the codes were extracted exactly from the points mentioned by the participants.

### Trustworthiness

In order to ensure the trustworthiness of the data, use was made of the criteria proposed by Guba and Lincoln (1994), i.e., credibility, transferability, confirmability, and dependability. In order to determine the credibility of the collected data, use was made of prolonged engagement (10 months). In addition, the codes, categories, and themes were continuously investigated and reviewed by the research team. The initial codes were also returned to the interviewees to be confirmed. In order to determine dependability, the research team was involved in the study process, and the results were presented to several external observers to explore the process of data analysis. In order to achieve confirmability, all research processes, particularly data collection and analysis, as well as the formation of the main themes, were approved by the external observers. Finally, maximum diversity was observed in the selection of the participants to enhance transferability.

## Results

This study was conducted on 22 older women with a mean age of 66.54 ± 5.76 years who suffered from urinary incontinence. Among the participants, 72% were married, and 28% were widowed. In addition, 9.18% of the participants had an academic education, 59.01% had diplomas and below diploma degrees, and 31.81% were illiterate. The participants had been suffering from urinary incontinence for 2–20 years.

The results revealed that the participants had various psychosocial experiences while living with and managing this disease, which affected their health and quality of life. Data analysis showed four main themes and 13 categories. The main themes included “problem incompatibility with the disease,” “mental impasse,” “facing social restrictions,” and “concealment and social escapism.” The main themes, categories, and the participants’ direct quotes have been presented in Table 1.

**TABLE 1:** Main themes, categories, and quotations identified through the interviews.

Main themes	Categories	Quotes
Problem in compatibility with the disease	- Helplessness against the disease - Problems in daily life - Dissatisfaction with living with the disease	- I feel that I can do nothing and I can’t control it - I’m helpless. I have done anything you can think of, but I haven’t been successful in - Wherever I am, if I reach the WC late, I get wet and I can’t control myself - This disease has disrupted my life. You have this problem at home and outside. It disturbs your daily life - It disrupts the normal life. You can’t live comfortably - Ever since I had this disorder, I haven’t been satisfied with my life - When people have this problem, they don’t enjoy their lives
Mental impasse	- Suffering due to the disease - Disappointment with the treatment - Feeling of shame - Continuous mental involvement with the disease - Self-blame - Fear, as a constant companion	- This disease destroys the patients’ mood and annoys them - I am suffering in my life. It bothers the patient - It disturbs the patient mentally. It bothers me a lot - I’m disappointed with the treatment - I did what was necessary, but it was not successful. I have lost hope in treatment - I feel ashamed because of suffering from this disease - I can’t tell my son that I have this problem and I have to undergo operation; I feel ashamed - When I attend a party, I feel ashamed because of going to WC frequently - You should be careful all the time, so that no problems will occur. These thoughts do not leave me alone even for a second - I think about this problem all the time. I always seek for ways to solve my problem - I always blame myself, because I didn’t care for myself when I was young and I put a lot of pressure on myself and now I have this problem - I’m sorry for myself, because I didn’t pay attention to my disease and didn’t do anything for its treatment - I’m afraid of going out or going to a party. I’m afraid of not controlling myself and losing my reputation - I’m afraid of being noticed by others - I’m afraid of being labeled inappropriately by others
Facing social restrictions	- Insufficient access to sanitation facilities in the society - Limitation of outdoor activities	- I always have problems for finding restrooms out of the house - There is no bathroom in the mosque and I can’t purify myself in case I get wet - I can’t find a place to change my wet clothes out of my house - I rarely go to mosques or other religious places. I’m afraid of not being able to control my urination - I haven’t gone on a trip for a long time, because there are few sanitation facilities on the roads and they are not clean - I don’t go to parties, I don’t go shopping
Concealment and social escapism	- Concealing the disease - Living a private life with the disease	- You can’t tell your family or friends about your disease. It is better to be kept secret - I tell myself that I have to keep this problem in my heart. I don’t like to talk about it with others - I don’t like others to be aware of my disease - I prefer to follow up my treatments alone - Each person has a problem in one’s life. I also have this problem and I have to take care of it. I shouldn’t engage others in this problem

### Problem Incompatibility With the Disease

The life experiences of older adults women with urinary incontinence showed their problems incompatibility with the disease. Despite using various strategies, they were not able to control their urination under sensitive circumstances. Therefore, they frequently faced failures in combating the disease and felt helpless in this regard. On the other hand, their inability to control their disease disrupted their daily lives, created numerous problems for them, and disturbed their tranquility, eventually leading to a feeling of dissatisfaction with life.

#### Helplessness Against the Disease

Despite using various strategies such as restricting the consumption of fluids, eating specific types of food, consuming medications, and reducing the activities that increased the intra-abdominal pressure, the participants were not able to control their disease under sensitive conditions including the times they were at parties, out of the house, or with others. This caused them to feel helpless in disease management. They also stated frequently that they were defeated by the disease.

*“When I suddenly sneeze or laugh at a party, the urine leaks unintentionally, and I cannot control it”* (P. 9).

*“I feel that I have been defeated. I have had enough. I feel helpless”* (P. 1).

#### Problems in Daily Life

This disease disrupted the patients’ everyday life processes, leading to an imbalance in their lives. In other words, the participants’ activities of daily living were affected by the disease.

*“This disease has affected my life. It has disturbed my tranquility”* (P. 8).

“*Ever since I had this problem, I have faced problems in doing my religious affairs because I can’t keep myself clean*” (P. 4).

*“From people’s perspective, this is not an acceptable disease. All people love cleanliness. If individuals are not clean or can’t keep themselves clean, their lives will be disrupted”* (P. 10).

#### Dissatisfaction With Living With the Disease

The limitations and problems associated with the disease resulted in the feeling of dissatisfaction with life among the participants. In other words, the participants frequently mentioned their dissatisfaction with living with this disease.

*“It has created many problems in my life. Living with this disease is like a nightmare. I do not enjoy my life. I am not satisfied with living with this disease”* (P. 4).

### Mental Impasse

While living with urinary incontinence, the participants suffered a lot. On the other hand, the ineffectiveness of their measures for managing the disease resulted in their disappointment with the treatment. Moreover, they felt ashamed and blamed themselves for suffering from the disease. They were also tired of thinking about the disease and controlling it all the time. Furthermore, they constantly feared while living with this disease, fear of being judged and rejected. These multifaceted mental pressures put them in a mental impasse.

#### Suffering Due to the Disease

In addition to the disease, the older participants suffered from getting wet frequently, frequent purification, and foul urine smell.

*“It is hard to tolerate the disease. It bothers the patient a lot”* (P. 12).

*“I am annoyed since I get wet frequently, and I need to purify myself”* (P. 5).

#### Disappointment With the Treatment

Frequent failures in the treatment and control of the disease and facing the peers’ negative experiences created a feeling of discouragement and disappointment among the older women.

*“I have done everything to treat the disease, but I was not successful. I have become disappointed”* (P. 15).

#### Feeling of Shame

Since the participants considered urinary incontinence a taboo, they felt ashamed of suffering from this disease. The experiences of unintentional urine leakage further stimulated this feeling at inappropriate times and places and in the presence of other individuals.

*“When you get wet in front of your family, friends, and even children, you feel ashamed”* (P. 3).

#### Self-Blame

The participants frequently blamed themselves for suffering from the disease. In this respect, they believed that the disease resulted from their lack of self-care. They also stated that the lack of timely follow-up of the disease caused the intensification of the condition.

*“When I get wet, I just blame myself because I did not care for the problem to solve it sooner”* (P. 18).

#### Continuous Mental Involvement With the Disease

The participants emphasized that they thought about their disease all the time. In other words, they lived with these mental ruminations. They believed that they had to be careful about their problem to manage it to the extent possible.

*“I think about my disease all the time. It does not get out of my mind even for a second. I have to be careful to avoid problems”* (P. 14).

#### Fear, as a Constant Companion

The participants lived with a constant fear resulting from their inability to control their urinations and the intensification of the disease over time.

*“I am afraid of the intensification of my conditions with aging”* (P. 11).

The participants were also frightened to unveil their disease because they feared facing their acquaintances’ inappropriate behaviors. They were also afraid of others’ judgments and attitudes toward the disease. They thought that their family and friends would not be willing to have relationships with them due to their disease.

*“I am afraid of changes in others’ behaviors, not being accepted by others. They may not be willing to have relationships with me. I am afraid of being rejected”* (P. 16).

### Facing Social Restrictions

The older women with urinary incontinence faced various social restrictions, including lack of access to sanitation facilities, which reduced their social activities.

#### Insufficient Access to Sanitation Facilities in the Society

The majority of the participants complained about the lack of sanitation facilities in the society. They maintained that they did not have access to public restrooms in some places. The inability to use Iranian toilets was yet another problem mentioned by the participants. Moreover, they did not have access to bathrooms, particularly in religious places, to purify themselves if necessary. They could not also find a place to change their wet clothes while they were out.

*“There are no public restrooms on many streets, and if there are, they are very dirty”* (P. 5).

*“When I get wet out of my house, I can’t find a safe place to clean myself and change my clothes”* (P. 8).

#### Limitation of Outdoor Activities

Considering the participants’ inability to control their urination and their insufficient access to sanitation facilities in society, they were obligated to limit their outdoor activities. Therefore, they avoided leaving the house or going to places without sanitation facilities to the extent possible. In other words, they only referred to places with public restrooms and had limited social activities.

*“I do not go anywhere to the extent possible. I leave the house only for work because I am afraid of not finding a public restroom”* (P. 19).

*“I have limited my recreational activities with my family and friends”* (P. 10).

### Concealment and Social Escapism

The study participants tended to conceal their disease. They emphasized that this condition was a personal problem, which caused them to escape from society to hide their disease.

#### Concealing the Disease

Although the disease had negative impacts on the participants’ social activities, mental health, and well-being, they were not willing to talk about their problem with their families, friends, and even the healthcare team due to the nature of the disease and in order to prevent social stigma. To hide their disease, some participants deprived themselves from forging social relationships with others and preferred to escape from society rather than unveiling their disease.

*“I have hidden my problem from my family and friends”* (P. 6).

*“I do not like to talk about it with others. I do not like others to be aware of my problem. I hide it from everyone”* (P. 17).

#### Living a Private Life With the Disease

The participants considered their disease a personal issue and avoided the engagement of others, even their family members, in their problem. They tended to manage and treat their disease on their own. They believed that talking to their family and friends about the disease did not help them solve the problem and resulted in sadness and sensitivity.

*“I consider this disease a personal problem. I do not like to involve my family in this issue”* (P. 18).

*“I tell myself that I have to shoulder the burden of this problem”* (P. 20).

## Discussion

This study aimed to determine older women’s experiences regarding the management of urinary incontinence. The findings demonstrated that the participants experienced numerous mental pressures and social restrictions, which were effective in their psychosocial health. Totally, four main themes were extracted from the data as follows: “problem incompatibility with the disease,” “mental impasse,” “facing social restrictions,” and “concealment and social escapism.”

Urinary incontinence is accompanied by psychosocial consequences in older adult women, which are sometimes even more harmful than physical consequences. This disease exerts vast effects on daily living activities, social interactions, perception of health status, and quality of life, eventually resulting in limitations whose degree depends on society’s cultural background. These limitations, in turn, affect women’s mental and social health. Previous studies also indicated that the incidence of psychosocial complications was associated with urinary incontinence. Accordingly, older people with urinary incontinence had a weaker psychosocial health status (Chiu et al., 2020; Omu et al., 2020; Lee et al., 2021).

Similarities seem to exist in the psychosocial meanings of urinary incontinence amongst older women living in different cultures, ethnic groups, and social conditions. However, this disease can be accompanied by more problems in societies with specific religious backgrounds. Evidence has indicated differences in disease management experiences of the older women living in such communities compared to those in other societies. For instance, in Islamic communities, urinary incontinence can have more negative psychosocial effects on older adults people due to the cultural-religious atmosphere ruling the society (Karlowicz, 2010; Avery and Stocks, 2016; Jaberi et al., 2019; Batmani et al., 2021). This disease has also been associated with being reserved, isolated, and low self-esteem among Muslim women (Jokhio et al., 2013).

In the present study, facing unexpected and uncontrollable disease conditions resulted in the participants’ helplessness and dissatisfaction with life due to disrupting their natural life processes. This was to some extent affected by the society’s sociocultural background and represented helplessness incompatibility with the disease, which led to numerous psychosocial consequences for the older adults. In the same line, Hägglund and Ahlström (2007) disclosed that the inability to control urination resulted in the feeling of incompetence in disease management and reduced self-esteem amongst older women with urinary incontinence. This eventually led to psychosocial problems like isolationism, anxiety, and depression (Hägglund and Ahlström, 2007). Consistently, Berges et al. (2014) and Kinsey et al. (2016) reported that urinary incontinence disrupted the patients’ activities of daily living, disturbed their normal life processes, and increased tensions in their lives. Other studies have also revealed dissatisfaction with life among older women with urinary incontinence. Accordingly, this disease negatively affected the patients’ quality of life (Sinclair and Ramsay, 2011; Cerruto et al., 2013; Saboia et al., 2017).

This disease causes psychological complications in older adults (Avery et al., 2013). Based on the current study findings, such complications as shame, fear, self-blame, disappointment, and constantly thinking about the disease put the older adults in a mental impasse, negatively affecting their mental well-being. The majority of studies in this field have also shown the feeling of shame among older patients with urinary incontinence (Hägglund and Wadensten, 2007; Nicolson et al., 2008). Moreover, patients considered this disease as a kind of stigma (Elstad et al., 2010). This attitude is frequently detected amongst Asian women, specifically South Asian ones, and affects discussions about the disease and reception of the related care services. On the other hand, concealing the inconveniences associated with the disease due to being shameful exerts a negative impact on patients’ mental health (Higa et al., 2008; Chiu et al., 2020). Furthermore, since urine is considered dirty in the Islamic culture and urinary incontinence is regarded as uncleanliness, older adults’ women with this disease have a constant fear from the way others may treat them. Previous studies also indicated that patients with urinary incontinence were constantly afraid of getting wet in front of others (Lim, 2016; Spencer et al., 2017), others’ behaviors and judgments about the problem (Avery et al., 2013), and being rejected by others (Andersson et al., 2008). In the same vein, Doshani et al. (2007) stated that these patients were afraid of losing their relationships with others due to their disease, which significantly impacted their mental health.

Self-blame is also among the psychological disorders detected in older people suffering from urinary incontinence (Senra and Pereira, 2015; Toye and Barker, 2020). Iranian women frequently sacrifice themselves, pay less attention to their health status, and prioritize other issues in their lives. This worsens their health problems, eventually leading to regret and self-blame. In the present research, the participants frequently blamed themselves for not having paid attention to the primary symptoms of the disease, not having taken timely measures for treating the disease and not having cared for themselves in this respect. Various studies have also revealed self-blame among patients with urinary incontinence, which was effective in their mental health (Mason et al., 1999; Teunissen et al., 2006; Avery et al., 2013; Senra and Pereira, 2015; Lee et al., 2021).

Other psychological problems mentioned in the current study included discouragement and disappointment of treatment, which affected their treatment process. Which agreed with the results of the research carried out by Nicolson et al. (2008). They found hopelessness together with anxiety and depression amongst patients with urinary incontinence. In their study, older women were also disappointed with urinary incontinence treatment and refused to seek treatment (Nicolson et al., 2008). In the current investigation, the participants were tired of thinking about controlling their disease under various circumstances. They could not stop thinking about the disease. Similarly, Hemachandra et al. (2009) reported that patients with urinary incontinence were tired of constantly thinking about the disease and controlling the conditions to avoid urine leakage, which harmed their mental health. Thus, the incidence of psychological disorders was considered an obstacle against mental well-being, accompanied by a mental burden among older women suffering from urinary incontinence (Sinclair and Ramsay, 2011; Avery et al., 2013).

The current study participants experienced significant social restrictions that affected their social functions and fulfillment of their social roles, eventually leading to social isolation. Therefore, it can be said that older people with urinary incontinence do not have a proper level of social health. Because social health encompasses individuals’ social skills, social function, and ability to consider themselves as a part of society. Therefore, the social health of older women with urinary incontinence is impaired due to their inability to interact effectively with others and society and their inability to establish satisfactory social relationships and play their social roles (Babanejad et al., 2013; Saeid et al., 2019).

Older people need social support and welfare facilities to participate in society and participate in social activities; therefore, lack of appropriate social support for older people with urinary incontinence and paucity of sanitation facilities in the society restrict the presence of these individuals in the society, disrupt the fulfillment of their social roles, and lead to social isolation, thereby affecting their social health (Avery et al., 2013). Other studies also demonstrated that insufficient social support for older people and lack of welfare amenities in society were among the main problems that reduced their social participation (Darvishpoor Kakhki et al., 2010; Saeid et al., 2019). On the other hand, when urinary incontinence is considered uncleanliness in a society with a specific cultural and religious background, purification facilities have to be sufficiently provided in the society so that older adults’ individuals with this disease can use them if necessary. Hence, in this study, the lack of these facilities in the society has been mentioned as a limitation for the presence of older adults in the community and for their performance of social activities. In this respect, Hosseini et al. (2021) stated in their study that lack of social support for older people and lack of the required facilities, decreased their presence in society and affected their health and well-being.

On the other hand, older adults’ involvement in social networks such as family, friends, and neighbor networks is considered a source of support, helping them achieve appropriate mental and social health and supplying their other health dimensions (Javanmardifard et al., 2020; Hosseini et al., 2021). However, although the older women in our study suffered from urinary incontinence and needed support in many dimensions, they were doubtful about talking to others, even the healthcare team and family, and were willing to conceal their disease and escape from society and social networks. They regarded the disease as a secret in their lives and insisted on hiding it from others and managing it on their own. Other studies also indicated that older people were doubtful about unveiling their disease, which implied their deprivation from receiving help to facilitate the disease management (Hemachandra et al., 2009; Elstad et al., 2010; Jokar et al., 2020). Thus, if older patients deprive themselves of taking part in these social networks due to their unwillingness to unveil their disease, their mental and social health will be affected (Avery et al., 2013).

Since the emphasis of older women in our study on escaping from society, hiding their disease, and depriving themselves from the existing support sources like family, friends, and healthcare team resulted in their escape from society, which led to social isolation and affected their social health. In this respect, Thomas and Liu (2017) referred to the importance of older people’s close relationships with their families, which helped them receive beneficial support and tolerate and manage their health problems. However, they stated that the participation of older adults in social networks decreased with age. Therefore, favorable family relationships play a more important role in their well-being and help them cope better with various stresses of this period and have healthier behaviors. This can, in turn, increase their self-esteem and exert a positive effect on their psychosocial health (Thomas and Liu, 2017).

Therefore, it is important to provide the facilities needed by these older adults in the community and also to provide conditions for the older adults with urinary incontinence to share their problems more easily with others, especially the family and health care team, to use the available support resources for better management of their disease; otherwise, it makes older women more exposed to the psychosocial effects of the disease.

## Conclusion

Even though urinary incontinence is not a life-threatening condition, it can considerably impact the mental and social health of older women suffering from the disease. These individuals face numerous mental and social challenges for their disease management, which affect their psychosocial well-being and quality of life. Paying attention to the psychosocial health of older adults with urinary incontinence based on the society’s cultural and religious background and providing them with psychosocial support on the part of the healthcare team, families, and friends can help them desirably manage their disease.

## Implications

Considering the growth of aging and the sociocultural background of the societies where older people live, it is necessary to pay attention to psychosocial health amongst older people suffering from urinary incontinence, examine these patients regarding the psychosocial complications of the disease, and find the origins of their problems. Besides, healthcare team members have to make genuine attempts to solve these problems. For instance, they are suggested to encourage such older patients to share their experiences with their peers, which can help break the associated taboos and promote treatment-seeking behaviors among these patients. Furthermore, the facilities needed by older adults’ individuals with urinary incontinence are recommended to be provided in society to be able to actively participate in the community. This will eventually affect their physical, mental, and social health and their quality of life.

## Data Availability Statement

The data analyzed in this study is subject to the following licenses/restrictions: The datasets used and analyzed during the current study are available from the corresponding author on reasonable request.

## Ethics Statement

The studies involving human participants were reviewed and approved by the Ethics Committee of Ahvaz Jundishapur University of Medical Sciences (IR.AJUMS.REC.1398.604, Proposal No. NCRCCD-9825). The patients/participants provided their written informed consent to participate in this study.

## Author Contributions

SJ, MG, FS, KZ, and FG were involved in the study conception and design, contributed to the data collection and analysis, critically revised the manuscript for important intellectual content, provided administrative and technical support, and supervised the work. SJ, MG, FS, and KZ drafted the manuscript. All authors contributed to preparing the manuscript and approved the submitted version.

## Conflict of Interest

The authors declare that the research was conducted in the absence of any commercial or financial relationships that could be construed as a potential conflict of interest.

## Publisher’s Note

All claims expressed in this article are solely those of the authors and do not necessarily represent those of their affiliated organizations, or those of the publisher, the editors and the reviewers. Any product that may be evaluated in this article, or claim that may be made by its manufacturer, is not guaranteed or endorsed by the publisher.
